# Continuous drainage for the treatment of gastric cancer with pericardial metastasis and cardiac tamponade: A case report

**DOI:** 10.1002/ccr3.7522

**Published:** 2023-06-13

**Authors:** Tadayuki Hirai, Wataru Omi, Yoichiro Nakagawa, Yusuke Shinjo, Yoshitaka Okabe, Chieko Kato, Takahiro Saeki, Satoru Sakagami

**Affiliations:** ^1^ Department of Cardiology National Hospital Organization Kanazawa Medical Center Kanazawa Japan

**Keywords:** cardiac tamponade, cardiology, gastric cancer, pericardiocentesis drainage, pericarditis

## Abstract

**Key Clinical Message:**

Signet‐ring cell gastric carcinomas presenting as pericardial effusion early in diagnosis are rare and associated with high mortality and a poor prognosis. There are two interesting aspects of this case: primary gastric carcinoma presenting as cardiac tamponade and the metastatic behavior of gastric signet‐ring cell carcinoma.

**Abstract:**

This report describes an 83‐year‐old man diagnosed to have cardiac tamponade due to massive pericardial effusion. A cytological analysis of the pericardial effusion disclosed adenocarcinoma. The patient was treated with continuous pericardial drainage and the amount of pericardial effusion decreased.

## Graphical abstract

Signet‐ring cell carcinoma detected in pericardial fluid.

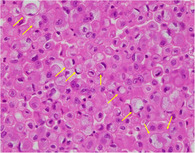

## 1 INTRODUCTION

In Japan, the aging population has been rapidly growing, and there have been more cases of gastric cancer. Pericardial metastasis is a relatively rare form of gastric cancer. In most reported cases, multiple metastases and invasion are seen, and local control of cardiac tamponade does not improve the prognosis.^1^ Cardiac tamponade is a heart disease with a fatal course that requires pericardial drainage as the primary treatment, and the timing and method are often difficult. Pericardial drainage plays an important role in diagnosis and is often considered early. This report showed carcinomatous pericarditis with a unique metastatic pattern that was successfully controlled by continuous pericardial drainage.

## 2 CASE PRESENTATION

**FIGURE 1:**
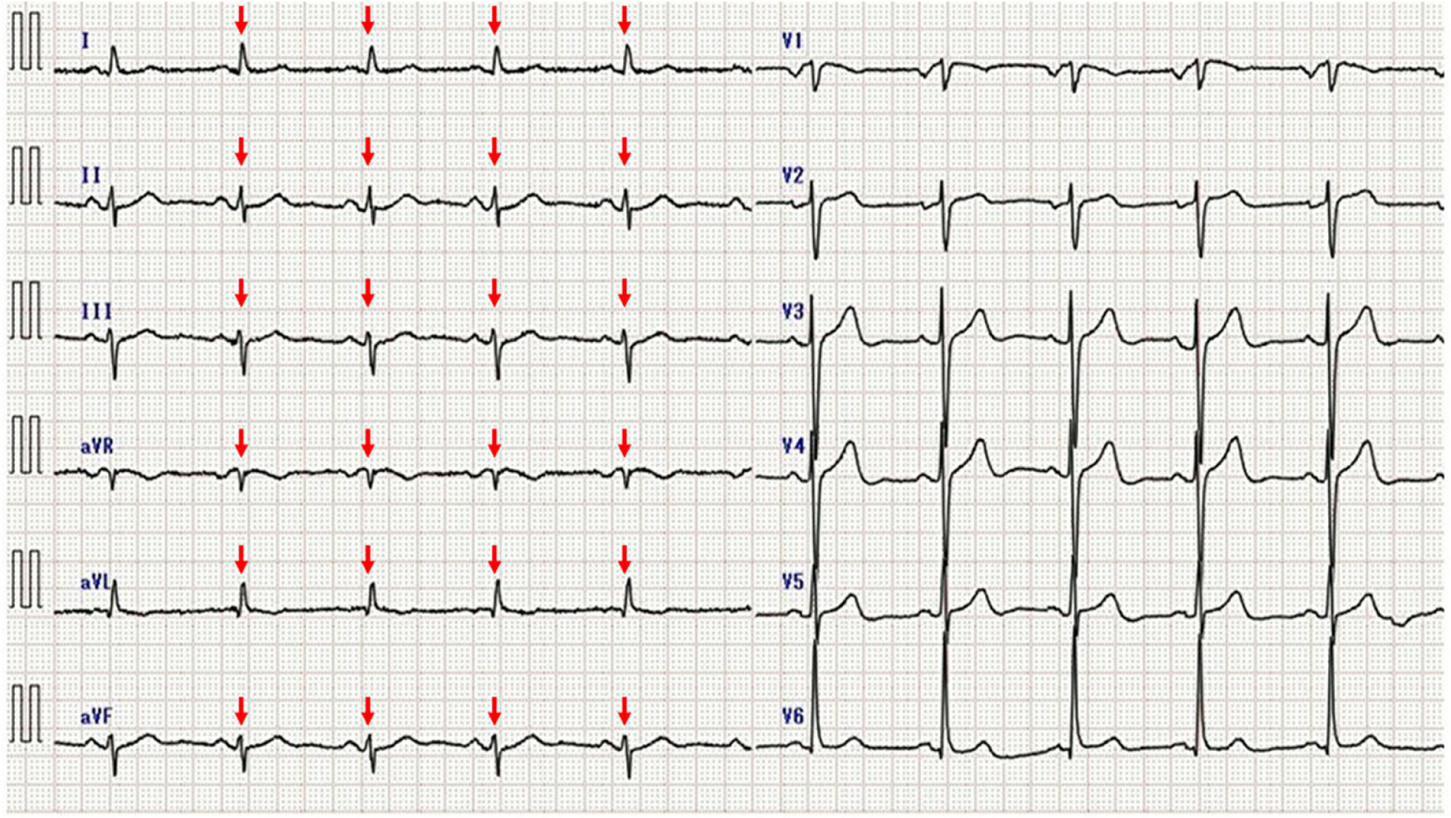
An electrocardiogram showed a normal sinus rhythm with low QRS voltage in precordial leads (red arrows).

**FIGURE 2:**
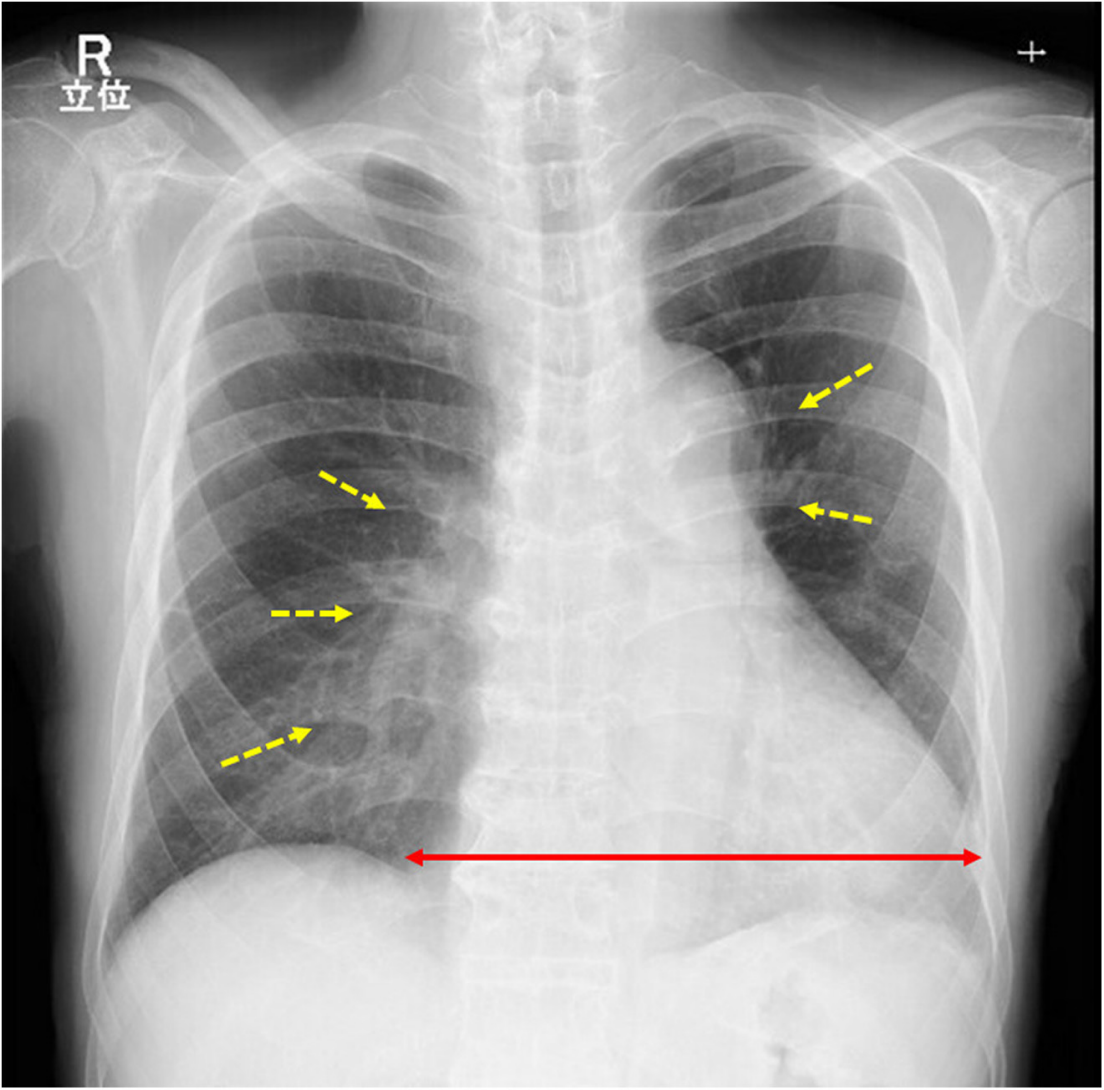
Erect Chest x‐ray showed cardiomegaly (two‐headed red arrow) and mild pulmonary congestion (yellow interrupted arrows).

**FIGURE 3:**
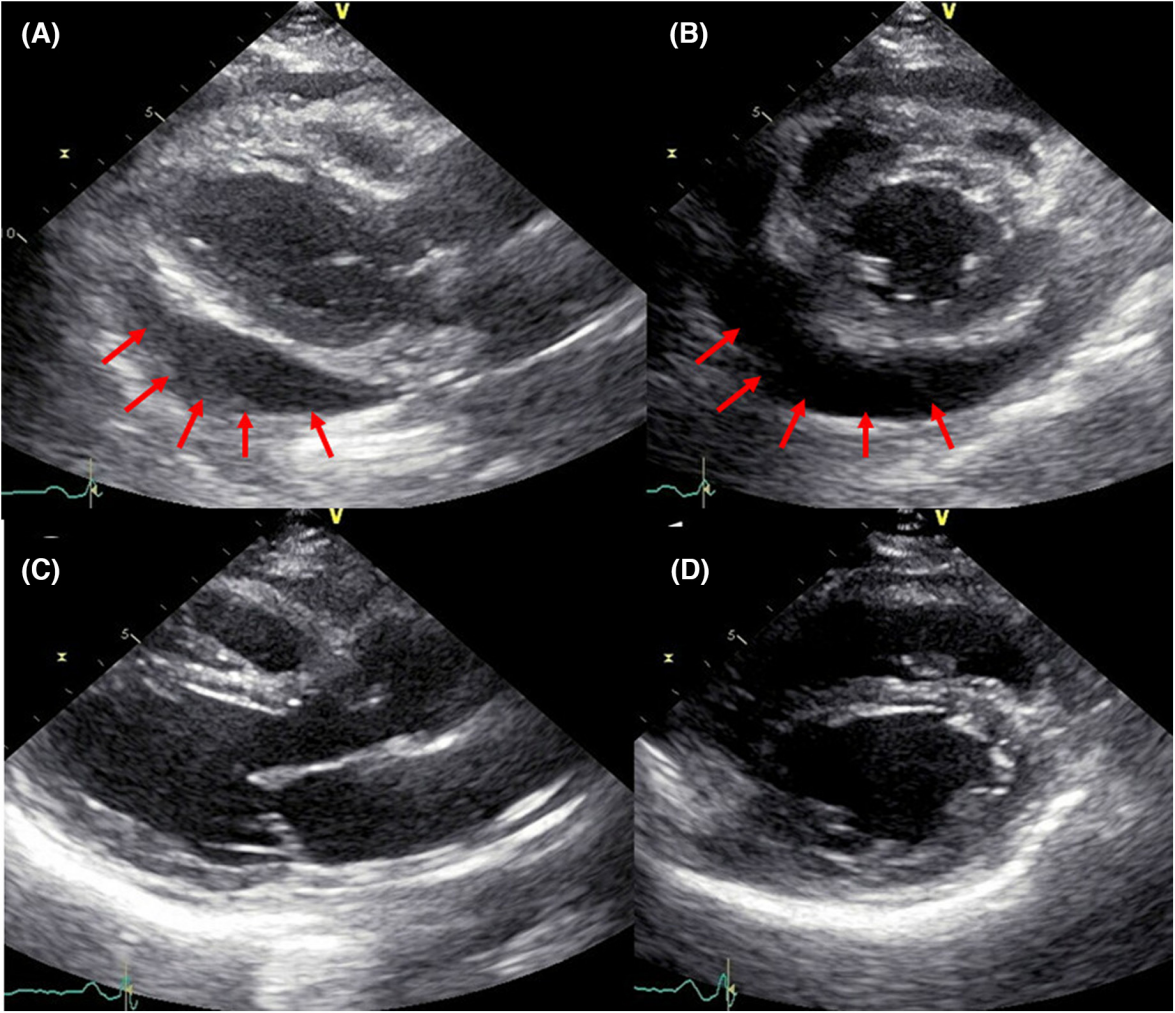
Echocardiography at the initial visit revealed pericardial effusion. (A) Parasternal long axis. (B) Parasternal short axis. Red arrows point to pericardial effusion. Echocardiography after pericardial drainage. Pericardial effusion remained low without excessive reaccumulation. (C) Parasternal long axis. (D) Parasternal short axis.

**FIGURE 4:**
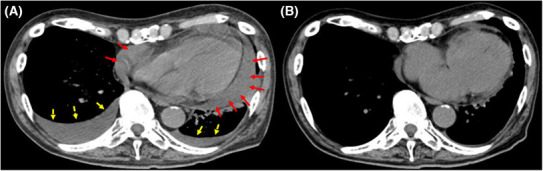
(A) Transaxial plain CT at the initial visit showed diffuse pericardial effusion (red arrows) and pleural effusion (yellow interrupted arrows). (B) CT after pericardial drainage. Pericardial effusion is clearly reduced.

**FIGURE 5:**
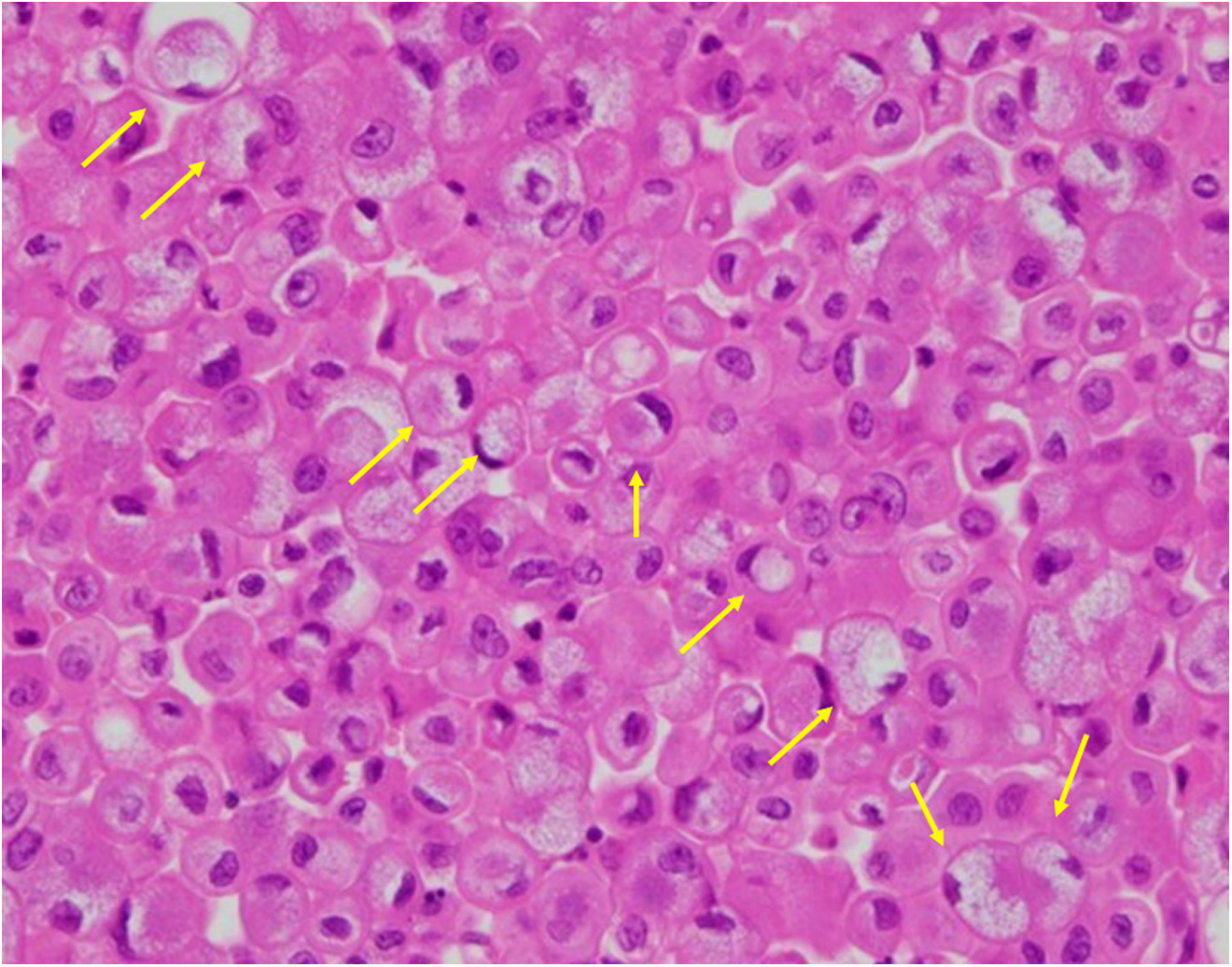
Gastric biopsy (PAS staining ×400) revealed signet‐ring cell cancer (yellow interrupted arrows).

An 83‐year‐old man was referred to secondary emergency hospital complaining of facial and lower limb with edema of 1‐month duration (Day 1). He had been an outpatient of his local clinic and was treated for dyslipidemia, hypertension, and Type 2 diabetes mellitus. He had suffered from facial and lower leg edema for 1 month before consultation. Although he complained of worsening edema, his vital signs were normal. The patient have a sort of shortness of breath on minimal exertion. The conjunctiva was not anemic nor icteric. Cervical lymph nodes were not palpable. Heart sounds were weak, and no cardiac murmur could be heard. The jugular vein was distended and respiratory sounds were clear. There were no abnormal findings in the abdomen. The liver and spleen were not enlarged and no focal mass was present. He had generalized edema, especially in the face and both lower legs. The patient could stay supine for a short period of time. Blood investigations on admission showed elevated levels of carbohydrate antigen 19‐9 (CA 19‐9), 174 U/mL (normal values, <37.0 U/mL) and brain natriuretic peptide (BNP), 88.4 pg/mL (normal values, <18.4 pg/mL). Twelve‐lead electrocardiogram (Figure 1) showed low voltage in the limb leads. Chest x‐ray (Figure 2) showed an enlarged cardiac shadow and a “sharp” bilateral costophrenic angle. Echocardiography (Figure 3A,B) showed pericardial effusion was present around the perimeter. An echo‐free space of 18 mm in the anterior surface of the right ventricle, 30 mm in the inferior wall of the left ventricle, and 19 mm in the posterior wall of the left ventricle was observed during systole. The right atrium and right ventricle collapsed during diastole. Altered blood filling and decreased heart output were suggested. Clinically, cardiac tamponade caused by a slow pericardial effusion caused by malignant tumor, collagen disease, and myocarditis has been considered. Contrast‐enhanced computed tomography of the abdomen (Figure 4A) showed a localized mass (42 × 51 mm) was found in the body of the stomach and pericardial effusion was noted. No lymph node swelling or abnormal shadow considered to be a metastatic mass was observed. Upper gastrointestinal endoscopy showed a huge and ulcer‐infiltrating tumor in the lower gastric body (Day 2). Gastric biopsy (Figure 5) showed microglandular structures were observed sporadically in poorly differentiated adenocarcinoma, and there were a few signet‐ring cell carcinomas with reduced connectivity and abundant intracellular mucus.

Hypotension and tachycardia were observed soon after admission, and transthoracic echocardiography showed increased pericardial effusion (Day 7). A pericardiocentesis was performed using an aseptic method on a subxiphoidal standard under the guidance of transthoracic echocardiography (Day 8). Regarding pericardiocentesis drainage, 600 mL of bloody effusion was aspirated in 30 min, and the drainage tube was removed. Cytological examination using pericardial effusion showed Class V (Papanicolaou classification), consistent with signet‐ring cell carcinomas. After the procedure, systemic edema and malaise dramatically improved. However, on the third day after drainage, pericardial effusion reappeared. Pericardiocentesis drainage was performed again (Day 13), and 380 mL of bloody pericardial fluid was aspirated in 30 min. At this time, the catheter (Argyle™ Blood Access LCV‐UK™ Catheter Kit) was left in situ and kept under negative pressure with a drainage bag (JVAC™ 100 cc Bulb Suction Reservoir). The amount of effusion gradually decreased and, having confirmed that effusion was not aspirated, the catheter was removed 1 week after the drainage (Day 18). The pericardial effusion remained small after catheter removal (Figures 3C,D and 4B). The patient was discharged, and chemotherapy was subsequently performed. The patient was diagnosed with gastric cancer stage IV. The performance status was good and pericardial effusion did not develop 5 weeks after removal of the pericardial drain. Soon after, chemotherapy was stopped at his request. He was offered the best supportive therapy. He died of cardiac arrest 1 month after best supportive care without severe cancer pain.

## 3 DISCUSSION

Pericardial effusion occurs in a variety of conditions.^2^ Causes of cardiac tamponade include idiopathic, malignancy, myocardial infarction, trauma, aortic dissection, uremia, infection, collagen diseases, amyloidosis, and vascular structural abnormalities such as hereditary hemorrhagic telangiectasia and arteriovenous malformation, coronary artery dissection, coronary artery aneurysm, coronary artery fistula, bleeding diathesis (such as blood disease), anticoagulants, and antiplatelet drugs. Thus, clinical course and images excluded specific causes and cytologic diagnosis finally identified malignant disease. In this case, the results of cytology of the pericardial fluid strongly suggested a connection with gastric cancer. Our final diagnosis, strengthened by the exclusion of other causes of cardiac tamponade, was gastric cancer with pericardial metastasis.

Metastatic cardiac tumors are apparent in 7.1% of autopsy patients with cancer, but they are rarely discovered while the patient is alive, and 90% remain asymptomatic.^3^ The incidence of cardiac metastasis varies depending on the tumors. Tumors showing a high incidence include pleural mesothelioma (48.4%), melanoma (27.8%), lung adenocarcinoma (21%), undifferentiated carcinoma (19.5%), lung squamous cell carcinoma (18.2%), and breast carcinoma (15.5%), ovarian carcinoma (10.3%), lymphomyeloproliferative neoplasm (9.4%), bronchoalveolar carcinoma (9.8%), renal carcinoma (7.3%), and pancreatic cancer (6.4%).^4^ Pericardial metastasis in gastric cancer is relatively rare (2.7–10%), and only 3.1% of patients develop cardiac tamponade.^5^ Nine cases of signet‐ring cell gastric carcinomas presenting as cardiac tamponade were found from earlier reported literature.^5^, ^6^, ^7^, ^8^, ^9^, ^10^, ^11^, ^12^, ^13^

There are three pathways of metastasis to the heart: direct invasion, through the bloodstream, and through the lymphatic system. The lymphatic system plays the major role in pericardial metastasis. Obstruction of lymphatic flow causes myocardial edema, systolic and diastolic dysfunction, which in turn exacerbates edema. The damage seen in these cases occurs due to lymphatic stasis and edema, which may increase the proliferation of tumor cells and metastases to other regions. The increased pressure may also destroy the wall of the lymph vessels, leading to the spread of stromal tumors.^4^

In this case, lymph node swelling was not observed by contrast CT. There were also no findings suggesting direct invasion of other surrounding organs, which leads to cardiac tamponade. This finding is uncommon; moreover, most previously reported “advanced” cases were characterized by multiple metastasis and invasion. The routes by which the cardiac metastases commonly travel from primary tumors are believed to be by direct extension, by lymphatic spread, by hematogenous spread, and by combinations of two or all three of the above. The progression of the primary tumor was so rapid that it had previously metastasized before it was large enough to form a primary mass. Histopathological findings showed signet‐ring cell carcinoma and it met the morphologically poorly differentiated adenocarcinoma.

Treatment and control of pericardial effusion is important in carcinomatous pericarditis. To our knowledge, there are no reports investigating the different therapeutic modalities available for the treatment of cardiac tamponade originating from gastric cancer. The patients with the longest survival were those treated by pericardial drainage, associated with palliative systemic treatment.^7^ Appropriate drainage may improve the prognosis, even in patients with carcinomatous pericarditis and cardiac tamponade.

Pericardial drainage should be considered in cardiac tamponade, but indwelling pericardial catheters have a success rate (defined as alleviation of tamponade and no need of further procedures) of 75% approximately.^14^ Repeated pericardiocentesis may increase the risk of constrictive pericarditis. If the pericardial effusion is difficult to control and the condition of the patient permits it, surgical treatment such as pericardial fenestration should be considered.^2^, ^15^ As previously reported, pericardial fenestration is one of the effective methods to prevent cardiac tamponade, but dissemination of cancerous pericardial fluid into the body cavity is a concern. In patients with localized pericardial metastasis, as in this case, the decision to perform pericardial fenestration is not straightforward, due to the possibility of promoting cancer dissemination and thus worsening the patient's prognosis. No randomized studies have compared the percutaneous drainage of pericardial effusions to the surgical drainage of pericardial effusions. Retrospective studies have shown that surgical drainage can reduce recurrence but increase the risk of peri‐procedure complications.^16^

The efficacy of intrapericardial injection of antineoplastic agents, such as bleomycin, for the treatment of carcinomatous pericarditis has been reported; this procedure is called sclerotherapy.^17^ Compared with simple pericardial drainage, less reaccumulation of pericardial effusion can be expected, but serious complications have also been reported, such as infections, constrictive pericarditis, and pericardial hemorrhage.

The echocardiographic findings suggested that pericardial adhesion was likely to prevent reaccumulation of effusion. Repair of the injured pericardial tissue (including blood and lymphatic vessels) that caused the effusion might be possible via compression, by exploiting the negative pressure of the pericardial sac. However, although this strategy could be effective in some cases, its reliability is not known. In such cases, close attention is needed because of the high risk of infection and fistula. To avoid infection and mechanical complications, pericardial catheters should not be left in place without any meaning, but we found no evidence of an increase in the frequency of complications over time.

In conclusion, this report showed advanced gastric cancer and cardiac tamponade with an uncommon metastatic pattern. This case illustrates successful diagnosis and treatment of the pericardial effusion with continuous drainage using a catheter and bag, which maintained negative pressure in the pericardial sac. This method may be useful in some cases of carcinomatous pericarditis.

## AUTHOR CONTRIBUTIONS

**Tadayuki Hirai:** Investigation; writing – original draft. **Wataru Omi:** Investigation; writing – review and editing. **Yoichiro Nakagawa:** Data curation. **Yusuke Shinjo:** Data curation. **Yoshitaka Okabe:** Data curation. **Chieko Kato:** Data curation. **Takahiro Saeki:** Investigation; writing – review and editing. **Satoru Sakagami:** Methodology; project administration; writing – review and editing.

## FUNDING INFORMATION

This report was not funded. There was no funding support for the case report.

## CONFLICT OF INTEREST STATEMENT

The authors have no conflict of interest to declare.

## ETHICS STATEMENT

This case report was derived from clinical practice and approved by the Institutional Ethics Committee at National Hospital Organization Kanazawa Medical Center, and informed consent for publication was obtained from the patient. All authors take responsibility for all aspects of the patient privacy and confidentiality.

## CONSENT

Following the patient's death, written informed consent was obtained from the patient's brother to publish the report.

## Data Availability

Data sharing not applicable to this article as no datasets were generated or analyzed during the current study.
